# Platinum-based neoadjuvant chemotherapy upregulates STING/IFN pathway expression and promotes TILs infiltration in NSCLC

**DOI:** 10.3389/fonc.2024.1346225

**Published:** 2024-02-15

**Authors:** Huan Gao, Xiaoni Zhang, Mengdi Ren, Aimin Jiang, Na Liu, Jingjing Wang, Xiaoqiang Zheng, Xuan Liang, Zhiping Ruan, Tao Tian, Xiao Fu, Yu Yao

**Affiliations:** ^1^ Department of Medical Oncology, The First Affiliated Hospital of Xi’an Jiaotong University, Xi’an, Shaanxi, China; ^2^ Department of Respiratory Medical, Xi’an International Medical Center Hospital, Xi’an, Shaanxi, China; ^3^ Institute for Stem Cell and Regenerative Medicine, The Second Affiliated Hospital of Xi’an Jiaotong University, Xi’an, China

**Keywords:** non-small cell lung cancer, neoadjuvant chemotherapy, STING, IFN, PDL1, tumor infiltrates T lymphocytes

## Abstract

**Objectives:**

To evaluate the effects of platinum-based neoadjuvant chemotherapy (NACT) on the STING/IFN pathway and tumor-infiltrating lymphocytes (TILs) in non-small cell lung cancer (NSCLC), as well as clinicopathological factors affecting patient survival.

**Materials and methods:**

A total of 68 patients aged 34-77 years with NSCLC who received neoadjuvant chemotherapy and surgical treatment from March 2012 to February 2019 were reviewed, and the clinical pathological data and paired tissue specimens before and after NACT were collected. Immunohistochemistry and immunofluorescence were used to detect the protein levels of STING, PD-L1 and IFN-β, and the infiltration density of CD3^+^ TILs and CD8^+^TILs. The correlation between the expression of STING, PD-L1, IFN-β and the infiltration density of CD3^+^ TILs and CD8^+^ TILs as well as the clinicopathological characteristics before and after NACT was analyzed. The relationship between the related indexes, clinicopathological features and prognosis was also discussed.

**Results:**

NACT increased the expression of STING, IFN-β and PD-L1 in tumor cells, and the infiltration of CD3^+^ and CD8^+^ TILs. In addition, ypTNM stage, ypN stage, changes in CD3^+^ TILs and in PD-L1 were associated with DFS (disease-free survival). CD3^+^ TILs changes and ypN stage were associated with OS (overall survival). Notably, ypN stage and CD3^+^ TILs changes were independent prognostic factors for DFS and OS.

**Conclusion:**

NACT stimulates STING/IFN-β pathway, promotes infiltration of CD3^+^ and CD8^+^ TILs, triggers innate and adaptive immunity, and also upregulates PD-L1, which complemented the rationale for neoadjuvant chemotherapy in combination with immunotherapy. In addition, DFS was longer in patients with ypTNM I, ypN0-1, and elevated CD3^+^TILs after NACT. Patients with ypN0 and elevated CD3^+^ TILs after NACT had better OS benefits.

## 1 Introduction

Immune checkpoint blockade (ICB) therapy has greatly advanced the clinical treatment of tumors. Chemotherapeutic agents such as cisplatin contribute to the efficacy of ICB drugs, but the specific molecular mechanisms remain unclear.

Cyclic GMP-AMP synthase (cGAS) is an innate immune receptor that recognizes cytoplasmic DNA from viruses, bacteria, mitochondria, and micronuclei. Upon binding cytoplasmic DNA, cGAS can activate the interferon gene stimulator (STING), thereby producing IFN-I. This process promotes dendritic cell maturation and migration, activates CD8^+^ T lymphocytes, and exerts cytotoxic effects. Thus, the cGAS/STING pathway plays an important role in the innate immune response against viruses and tumors (1, 2). Genomic instability, mutations or deletions of tumor suppressor genes, oxidative stress, and metabolic disorders can lead to the destruction of nuclear and mitochondrial DNA in tumor cells. Ultimately, this can appear in the form of micronuclei, chromatin fragments, or free telomere DNA, which can cause cytoplasmic leakage of DNA (3). Apart from intrinsic chromosomal instability, DNA damage and micronucleus formation can also be induced by chemotherapeutic drugs and radiation therapy. IFN-I has a crucial role in anti-tumor immunity by linking innate immunity to adaptive immunity, including the influx of T cells (4).

PD-L1 produced by tumor cells binds with PD-1 on lymphocytes, suppressing activation of cytotoxic T-lymphocytes and reducing anti-tumor effects. The effectiveness of immunotherapy can be impacted by PD-L1, tumor immunogenicity, tumor-infiltrating lymphocytes, immunosuppressive cells, and cytokines present in the tumor microenvironment (5). Therefore, identifying relevant indicators to predict immunotherapy effectiveness, optimizing patient populations, and maximizing clinical benefits are important steps to take. Research has demonstrated that neoadjuvant chemotherapy (NACT) can increase PD-L1 expression by activating immune-related pathways, such as IFN, STAT3, and TNF-α (6).

Platinum is the basic cytotoxic agent for chemotherapy regimens, which can induce DNA damage. Currently, the impact of neoadjuvant chemotherapy on the cGAS/STING pathway as well as downstream molecules remains to be complemented. Our study compared the expression changes of PD-L1, STING, IFN-β, CD3^+^ TILs and CD8^+^ TILs in paired tissues pre- and post- NACT treatment, as well as the relationship between clinicopathological factors and survival. In summary, our study revealed that platinum-based NACT has the ability to alter certain immune components within the tumor microenvironment (TME) and modulate the immune status, provides clinical evidence to support chemosensitization immunotherapy.

## 2 Materials and methods

### 2.1 Patient cohort

A total of 68 patients aged 34-77 years with non-small cell lung cancer who received neoadjuvant chemotherapy and surgery in the First Affiliated Hospital of Xi ‘an Jiaotong University from March 2012 to February 2019 were selected. Paraffin sections and clinical records were retrospectively retrieved. The patients were monitored for disease progression. The study was approved by the Ethics Committee of the First Affiliated Hospital of Xi ‘an Jiaotong University (NO. XJTU1AF2021LSK-256).

### 2.2 Immunohistochemistry and immunofluorescence

Pre- and post-treatment tissue specimens were stained for STING (Cell signaling technology, #13647S, 1:100), PD-L1 (Bioss, bs-1103R, 1:300), IFN-β (Bioss, bs-23731R, 1:300), CD3 (Proteintech, 17617-1-AP, 1:100), CD8 (Proteintech, 66868-1-Ig, 1:1000). 5-um-thick tissue sections were deparaffinized in xylene, passed through graded alcohols, and antigen repaired with citrate buffer (pH=7.6) in a steam pressure cooker. The sections were blocked with 5% BSA 1H and incubated with primary antibodies at 4°C overnight, and then washed in 50 mM Tris-HCl, pH 7.4 and incubated with horseradish peroxidase-conjugated secondary antibodies. Immunoperoxidase staining was performed with DAB system. Slides were counterstained with haematoxylin. Immunofluorescence-stained sections were added dropwise with DAPI, incubated for 10 minutes and then rinsed 3 times with PBS. Immunohistochemical sections were dehydrated in in graded alcohol and xylene, and sealed with neutral gum. Immunostaining sections were sealed with glycerin.

### 2.3 IHC and IF staining quantification

Microscopically, STING and IFN-β were localized in the cytoplasm, and PD-L1, CD3 and CD8 were localized in the cell membrane. Five high-power fields were randomly selected to calculate the percentage of positive tumor cells. The pathologist was blinded to the patient information. IFN-β and STING positive cells were divided into 5 levels according to percentage (score 0, ≤5%; score 1, 6-25%; score 2, 26-50%, score 3, 51-75% and score 4, 76-100%). There are 4 levels (-, score 0; +, score 1; ++, score 2; +++, score 3) based on staining intensity. Analysis was performed using the product of the positive cells score and the staining intensity score. PD-L1 positive level was divided into 3 grades according to the TPS score: <1%, score 0; 1%-49%, score 1; ≥50%, score 2. CD3 and CD8 were stained on the cell membrane. Five high magnification fields were randomly selected and the percentage of positive cells was calculated. Slides were scanned using the Pannoramic DESK (3DHISTECH).

### 2.4 Chemotherapy response evaluation

The clinical response to NACT was assessed according to the Response Evaluation Criteria in Solid Tumors 1.1. The degree of pathological response was evaluated based on the proportion of microscopic surviving tumor cells in the tissue sections after the NACT. No pathological response: residual tumor cells > 10%; Major pathological response (MPR): residual tumor cells ≤ 10%; Complete Pathological Response (pCR): No residual tumor cells.

### 2.5 Statistical analysis

Statistical analyses were performed with the SPSS 22.0, Sangerbox 3.0 (http://www.sangerbox.com/tool) and Graphpad Prism 6. Spearman correlation analysis was used to test the associations between indicators. The Wilcoxon rank sum test was used to analyze the differences in IHC, IF signals before and after NACT. Cumulative survival rate was performed using the Kaplan-Meire method, and analyzed by log-rank test. Cox proportional models were used to determine the hazard ratios among patients in the different groups. All tests were evaluated with a 95% confidence interval. *P* values <0.05 were considered statistically significant.

## 3 Results

### 3.1 Patient characteristics

Sixty-eight cases of NSCLC with high-quality FFPE tissue and clinicopathological information available were included in the study (**Table 1**). The median age of the patients was 59.5 years (range, 34-77 years). Males and smokers were in the majority (82.3% and 85.5%, respectively). Histological type of squamous carcinoma in 69.4% of cases in the cohort and adenocarcinoma in 30.6%. Thirty-three (48.4%) subjects were moderately differentiated and the remainder were poorly differentiated. All patients received at least two cycles of platinum-based NACT. All 68 pairs of specimens obtained were matched with pre- and post-NACT tissues.

**Table 1:** clinical characteristics of the patient cohort.

Characteristic	N(%)
No. of patients	62
Gender	
Male	51 (82.3)
Female	11 (17.7)
Median age (years)	59.5
ECOG PS	
0	28 (45.2)
1	34 (54.8)
Smoking	
Yes	53 (85.5)
No	9 (14.5)
Smoking index	
≤ 400	23 (37.1)
> 400	39 (62.9)
Comorbidity	
Yes	24 (38.7)
No	38 (61.3)
Pathological types	
Adenocarcinoma	19 (30.6)
Squamous carcinoma	43 (69.4)
Pathological differentiation	
Moderately	30 (48.4)
Poor	32 (51.6)
cTNM	
IB	7 (11.3)
II	17 (27.4)
III	38 (61.3)
cT	
T1	4 (6.5)
T2	26 (41.9)
T3	17 (27.4)
T4	15 (24.2)
cN	
N0	13 (21.0)
N1	24 (38.7)
N2	25 (40.3)
Imaging evaluation	
CR+PR	44 (71.0)
SD	16 (25.8)
PD	2 (3.2)
ypTNM	
0	3 (4.8)
I	15 (24.2)
II	27 (43.5)
III	17 (27.4)
ypT	
ypT0	3 (4.8)
ypT1	16 (25.8)
ypT2	26 (41.9)
ypT3	10 (16.1)
ypT4	7 (11.3)
ypN	
ypN0	35 (56.5)
ypN1	15 (24.2)
ypN2	12 (19.4)
Resection quality	
R0	57 (91.9)
R1	5 (8.1)
Pathological response*45 cases (72.6%)	
pCR + MPR	11 (17.7)
Not reach MPR	34 (54.8)
Pathological downstaging (global)	
Yes	41 (66.1)
No	21 (33.9)
Neoadjuvant chemotherapy regime	
TP	14 (22.6)
DP	26 (41.9)
GP	15 (24.2)
PP	7 (11.3)
Time from end of chemotherapy to operation (week)	
≤ 4	26 (41.9)
> 4	36 (58.1)
Adjuvant chemotherapy	
Yes	48 (77.4)
No	14 (22.6)

ECOG PS, Eastern Cooperative Oncology Group performance status; TP, paclitaxel plus cisplatin; DP, docetaxel plus cisplatin; GP, gemcitabine plus cisplatin; PP, pemetrexed plus cisplatin.

### 3.2 Upregulation of STING, IFN-β, PD-L1 expression and increased infiltration of CD3^+^ and CD8^+^ TILs after NACT

**Figure 1:**
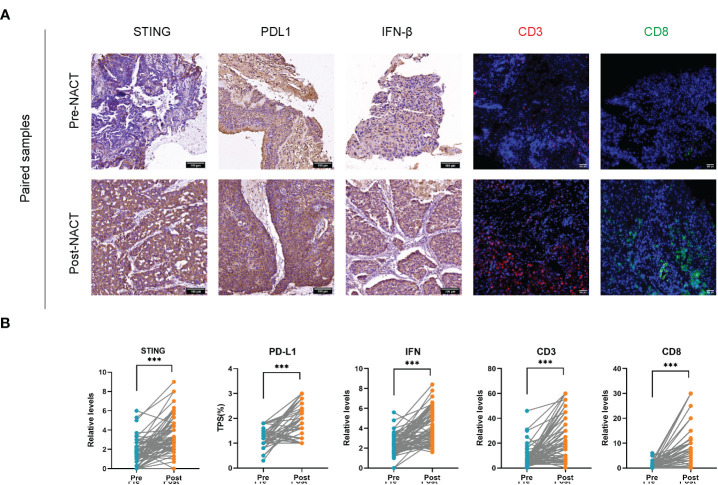
Upregulation of STING, IFN-β, PD-L1 expression and increased infiltration of CD3^+^ and CD8^+^ TILs after neoadjuvant chemotherapy (NACT). **(A)** Immunohistochemistry (IHC) images of STING, PDL1, IFN-β and Immunofluorescence (IF) images of CD3^+^, CD8^+^ in paired pre-NACT and post-NACT lung cancer samples. Scale bar, 100 μm. **(B)** Relative levels of IHC score and IF density in paired pre-NACT (n = 68) and post-NACT (n = 68) lung cancer tissues. *** *P* < 0.001.

It is reasonably hypothesized that DNA damaged by platinum-based chemotherapeutic agents triggers innate and adaptive immunity by binding to cGAS, activating STING located in the endoplasmic reticulum, and promoting IFN-β secretion and CD8^+^ TILs activation. Therefore, this study performed IHC on 68 cases of paired tissue sections pre- and post-NACT. The results are showed in **Figures 1A, B**. Consistent with speculation, the IHC score of STING was significantly higher after NACT compared to pre-NACT (pre- vs. post-: 1.57 vs. 3.42, *P* < 0.001). Similar results were obtained for IFN-β (pre- vs. post-: 2.34 vs. 4.26, *P* < 0.001). PD-L1 expression was also measured, and significantly higher after NACT compared to before (pre- vs. post-: 1.15 vs. 1.81, *P <*0.001).

In addition, we also evaluated the infiltration of CD3^+^ and CD8^+^ TILs. The proportion of CD3^+^ TILs was significantly higher in post-NACT samples compared to pre-NACT ones (pre- vs. post-: 7.89% vs. 23.91%, *P <*0.001). Similar results were obtained for CD8^+^ TILs (pre- vs. post-: 0.68% vs. 8.60%, *P <*0.001). These data suggested that NACT leads to upregulation of STING, IFN-β, PD-L1 expression and increased infiltration of CD3^+^ TILs and CD8^+^ TILs.

### 3.3 Association of STING, IFN-β, PD-L1, CD3^+^ and CD8^+^ TILs with DFS and OS

**Figure 2:**
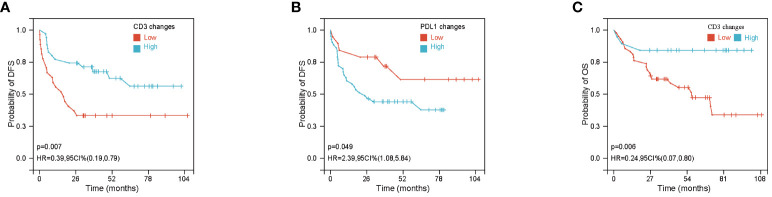
Association of CD3^+^ and PDL1 changes with disease free survival (DFS) or overall survival (OS). **(A, B)** Kaplan–Meier Curves of DFS based on CD3^+^ density **(A)** and PDL1 IHC score **(B)** changes after neoadjuvant chemotherapy (NACT). **(C)** Kaplan–Meier Curves of OS based on CD3^+^ changes after NACT.

After finding that STING, IFN-β, PD-L1, CD3^+^ and CD8^+^ TILs were all increased after NACT, we further analyzed the relationship between changes in these indicators and DFS. Among the 68 patients, 62 patients have complete follow-up data. The optimal cut-off values of STING, IFN-β, PD-L1, CD3 and CD8 fold change were calculated using the R package maxstata. We found that patients in CD3 fold change-high group have a longer DFS (*P* = 0.007) (**Figure 2A**). Moreover, PDL1 fold change-low group also predict longer DFS (*P* = 0.049) (**Figure 2B**). Meanwhile, OS of CD3-high group patients were also significantly better than that of CD3-low group patients (*P* = 0.006) (**Figure 2C**). The remaining factors not significantly different from DFS and OS were presented in **Supplementary Figure S1**.

### 3.4 ypTNM, ypN stage and CD3 changes were independent prognostic factors for DFS, ypN stage and CD3 changes were independent prognostic factor for OS

**Figure 3:**
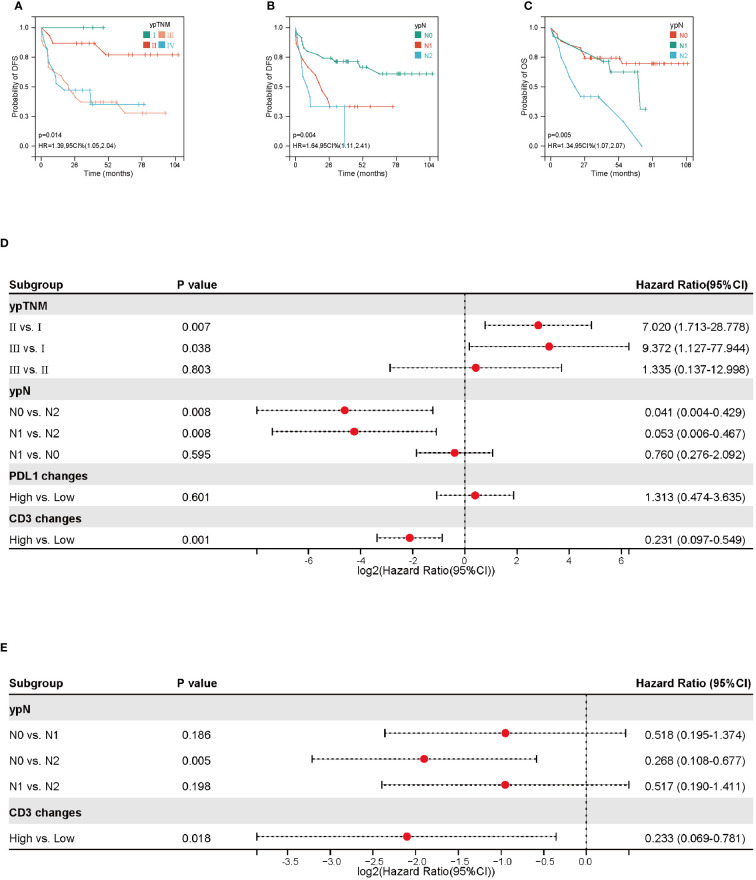
Prognostic factors for disease free survival (DFS) and overall survival (OS). **(A, B)** Kaplan-Meier curves of DFS based on ypTNM and ypN stage. **(C)** Kaplan-Meier curves of OS based on ypN stage. **(D)** Forest plot of DFS according to ypTNM, ypN, PDL1 IHC changes and CD3^+^ density changes. **(E)** Forest plot of OS according to ypN and CD3^+^ density changes.

**Figure 4:**
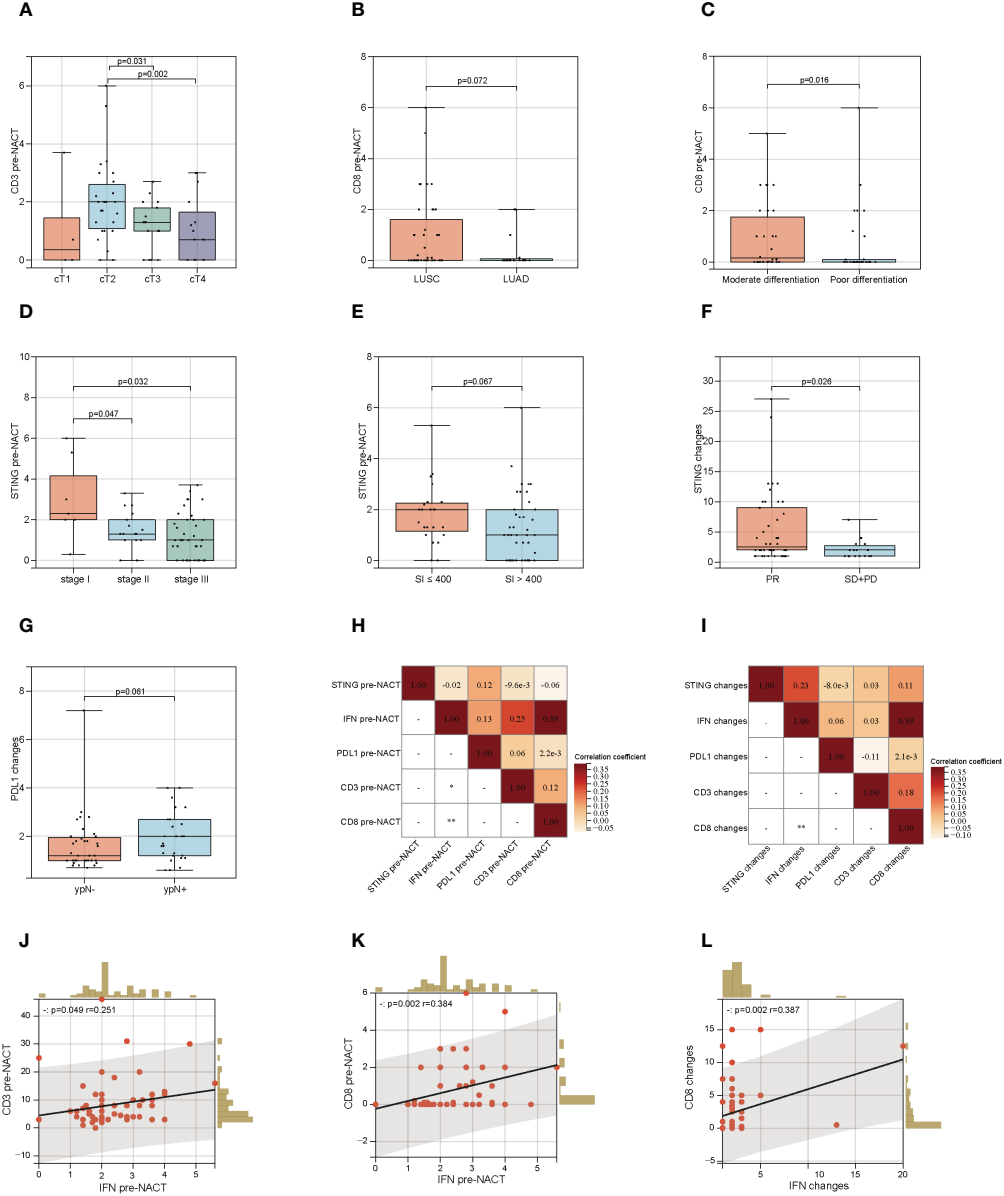
Association of STING, PDL1, CD3^+^ and CD8^+^ TILs with clinicopathological information. **(A)** Association of CD3^+^ TILs density pre-neoadjuvant chemotherapy (NACT) with cT stage. **(B)**Association of CD8^+^ TILs density pre-NACT with pathological types. **(C)**Association of CD8^+^ TILs density pre-NACT with pathological differentiation. **(D)** Association of STING IHC score pre-NACT with cTNM stage. **(E)** Association of STING IHC score pre-NACT with smoking index. **(F)** Association of STING IHC score changes after NACT with Imaging evaluation. **(G)** Association of PDL1 IHC score changes after NACT with ypN. **(H)** Correlation analysis based on data matrix for STING, IFN-β, PD-L1 IHC score and CD3^+^, CD8^+^ density pre-NACT. **(I)** Correlation analysis based on data matrix for STING, IFN-β, PD-L1 IHC score and CD3^+^, CD8^+^ density changes post-NACT. **(J)** Scatter plot of correlation between IFN IHC score and CD8^+^ density pre-NACT. **(K)** Scatter plot of correlation between IFN IHC score and CD3^+^ density pre-NACT. **(L)** Scatter plot of correlation between IFN IHC score and CD3^+^ density changes post-NACT. * *P* < 0.05, ** *P* < 0.01.

We performed an exploratory analysis of independent prognostic factors that may influence the efficacy of NACT for NSCLC. Based on univariate analysis, there was a statistically significant positive correlation between DFS and ypTNM stage, ypN stage, CD3 fold changes and PD-L1 fold changes (ypTNM stage and ypN stage are shown in **Figures 3A, B**, the rest are shown in **Figure 2**). There was no significant difference in DFS and OS among other clinicopathological factors, as shown in **Supplementary Figures S1**, **S2**. COX proportional-hazards model showed ypTNM stage, ypN stage and CD3 fold changes had independent prognostic significance in DFS (**Figure 3D**). At the same time, we evaluated the relevant indicators that affect OS. Univariate analysis showed that OS was also significantly correlated with ypN stage and CD3 fold changes (ypN stage was shown in **Figure 3C**, CD3 fold changes has shown in **Figure 2**). COX proportional-hazards model showed that ypN stage and CD3 fold changes were independent prognostic factors for OS (**Figure 4E**). It is worth noting that despite statistically insignificant differences, DFS appeared to be longer in patients with squamous carcinoma and those who achieved pathological remission, and OS was longer in patients who achieved pathological remission (**Figures S2A–C**).

### 3.5 Association of STING, IFN-β, PD-L1, CD3^+^ and CD8^+^ TILs with clinicopathological features

We finally explored whether STING, IFN-β, PD-L1, CD3 and CD8 correlated with clinicopathological factors. As shown in **Figure 4A**, in baseline level, CD3^+^ TILs were correlated with cT stage. cT2 stage group seemed to have a higher CD3^+^ TILs infiltration (cT2 vs. cT3, *P*=0.031; cT2 vs. cT4, *P*=0.002). CD8^+^ T cell was related with pathological types and differentiation. The moderately differentiated and LUSC group had higher CD8^+^ TILs infiltration (LUSC vs. LUAD, *P*=0.072; moderately differentiation vs. moderately differentiation, *P*=0.016) (**Figures 4B, C**). STING pre-NACT correlated with cTNM stage and smoking index. STING was higher in the SI<=400, cTNM I stage group (SI<=400 vs. SI>400, *P*=0.067; Stage I vs. Stage II, *P*=0.047; Stage I vs. Stage III, *P*=0.032) (**Figures 4D, E**).

As shown in **Figures 4F, G**, we also found that STING fold changes after NACT were associated with imaging evaluation. Compared to SD+PD patients, STING fold changes were higher in PR group, *P* = 0.026. Meanwhile, PDL1 fold changes were seemed higher in ypN^+^ group, *P* = 0.061. In addition, correlation analysis based on data matrix for STING, IFN-β, PD-L1, CD3 and CD8 at baseline level revealed that IFN-β positive correlated with CD3 (r=0.251, *P* =0.049) and CD8 (r=0.384, *P* =0.002) (**Figures 4H, J, K**). After NACT treatment, IFN-β fold changes were also positively correlated with CD8 fold changes (r=0.387, *P* =0.002) (**Figures 4I, L**).

## 4 Discussion

cGAS-STING, also known as TMEM173, ERIS, MITA, or MPYS, was originally found to be a receptor for cGAMP anchored to the endoplasmic reticulum. When damaged DNA is present in the cytoplasm, STING is activated, which promotes the formation of cGAMP. Upon binding to cGAMP, STING undergoes conformational changes, migrating from ER to the Golgi apparatus, and finally to the perinuclear microsomes, where it mobilizes downstream molecules to induce inflammatory responses. The cGAS-STING pathway is involved in the innate immune response and plays an important role in the resistance to pathogens (7).

Chromosomal instability is a significant feature of tumor cells. The unstable chromosomes form micronuclei after mitosis, which rupture frequently in the S phase, allowing dsDNA to leak into the cytoplasm and activating the cGAS-STING pathway. In addition to the intrinsic chromosomal instability, chemotherapeutic drugs and radiation that can destroy DNA can induce DNA damage and micronucleus formation, leading to activation of the cGAS-STING pathway and inflammatory responses. In addition, the damaged DNA can also act on relevant immune cells of TME to activate the cGAS-STING pathway (8).

Platinum inhibits DNA replication and induces DNA damage by forming cross-links with the DNA of tumor cells. The damaged dsDNA activates the cGAS-STING pathway to promote IFN-β expression, while IFN-β can upregulate tumor cell expression of PD-L1, increase CD3^+^ TILs and CD8^+^ TILs infiltration, and initiate adaptive immune responses (9–11). The role of cGAS-STING pathway in NSCLC and its prognostic significance are still being explored. Consistent with our findings, Della et al. found that cisplatin treatment increased STING and PD-L1 expression in tumor tissues, and further found that STING expression was associated with gene mutation status, with lower levels of STING expression in NSCLC with STK11 mutations and high levels of STING and immune-related gene in NSCLC with TP53 mutations expression (12).

The carcinogenic substances such as nicotine and benzopyran in tobacco can damage lung tissue and promote the occurrence of lung cancer. Smoking intensity and smoking duration are the risk factors affecting the prognosis of lung cancer. The study found that smoking can affect the chemotherapy effect of patients with lung cancer. On one hand, nicotine and other components in cigarettes can inhibit the killing ability of cisplatin to tumor cells by reducing the blood concentration of chemotherapy drugs. On the other hand, nicotine can promote the synthesis of anti-apoptotic protein Bcl-2 and inhibit the production of apoptotic protein, thus promoting DNA synthesis and cell proliferation, reducing DNA damage induced by cisplatin and increasing drug resistance of tumor cells (13). Our research found that STING protein level before NACT was related to smoking index. Combined with the previous research results, we speculate that it may be because smoking inhibits the DNA damage of tumor cells induced by platinum chemotherapy drugs, weakens the activity of cGAS-STING pathway and reduces the level of STING protein. In addition, the low level of STING expression is associated with the poor prognosis of NSCLC patients (14). Consistent, in our study, patients with PR and earlier TMN stage have higher STING expression levels. After neoadjuvant chemotherapy, patients with more significant STING increase have longer median DFS. Therefore, we believe that the activation of cGAS-STING pathway may improves the prognosis of NSCLC patients.

In a systematic literature review, 10 studies showed that high PD-L1 expression was associated with a poorer prognosis (15). There are also findings that PD-L1 expression is not associated with prognosis in NSCLC (16). In this study, we found increased levels of PD-L1 protein after neoadjuvant chemotherapy and a worse prognosis for patients in the group with elevated PD-L1.

Immune-related cells and immunosuppressive cells involved in the anti-tumor immune response in TME play an important role in the development of tumors. The depletion of T cells in TME weakens the anti-tumor effect of the body, while cytotoxic chemotherapeutic agents can induce the production of tumor neoantigens and activate the immune response. In a study of melanoma, the STING signaling pathway was found to increase MHC expression, promote IFN-γ expression and infiltration of TILs, while in STING-deficient melanoma, it attenuates the recognition of TILs to tumor cells and mediates resistance to immunotherapy (17). Studies have reported that pathological tissue sections from patients with a large infiltration of TILs in breast cancer TME after neoadjuvant chemotherapy reached pCR without detecting surviving tumor cells under the microscope (18). In our study, the infiltration of CD3^+^ TILs and CD8^+^ TILs increased after neoadjuvant chemotherapy, which is consistent with the results of previous studies. And it was found that before NACT, the infiltration density of CD8+ TILs were higher in LUSC compared to LUAD. This is consistent with a previous report by Chen et al. who suggested that the infiltration density of CD8^+^ TILs was significantly increased in squamous lung cancer compared to lung adenocarcinoma (19).

TILs have a significant impact on the prognosis of tumors (20). Intratumoral infiltration of CD4^+^ and CD8^+^ T cells has been found to be independently associated with good clinical outcomes (21). According to a large clinical trial, CD8^+^ T cell infiltration in breast cancer is associated with a reduced relative risk of death from the disease, and tumor lymphocyte infiltration improves risk stratification in patients with ER-negative and ER-positive HER2-positive subtypes of breast cancer (22). Mao et al. also found that infiltration of CD8^+^ TILs was associated with good DFS in breast cancer (23). Ding et al. assessed the prognostic value of TILs in liver cancer by meta-analysis and found that infiltration of CD3^+^ TILs and CD8^+^ TILs was significantly associated with improved survival (24). In NSCLC, it has been found that patients with concurrent infiltration of CD8+ T cells and CD4+ T cells have higher survival rates (25). In this study, we investigated the correlation between changes in CD3^+^ and CD8^+^ TILs infiltration levels before and after NACT and DFS, and our findings showed that increased infiltration of CD3^+^ and CD8^+^ TILs after NACT is a protective factor for DFS.

ICIs have been approved for routine clinical treatment of many cancers. For patients with NSCLC with positive PD-L1 expression, ICIs can be used as first-line therapy. However, only 20-30% of NSCLC are sensitive to ICIs, and most patients have a low response to immunotherapy and are prone to drug resistance, which affects the treatment outcome. The efficacy of immunotherapy is highly dependent on PD-L1 expression status, tumor immunogenicity, the abundance of antigen expression within the tumor, and immune cell infiltration. The cGAS-STING pathway, by sensing damaged cytoplasmic DNA, activates DCs, promotes IFN-I secretion, upregulates PD-L1 expression on tumor cell surface, promotes antigen presentation by APC, increases CD8^+^ T lymphocyte infiltration, regulates TME, and connects innate and cellular immunity. Therefore, combining STING agonists with immunotherapy may increase the sensitivity of immunotherapy, transform immune “cold tumor” into “hot tumor”, improve the immunogenicity of tumor, and enhance the body’s ability to kill tumor. Study has reported a STING agonist MK-1454 exhibited robust tumor cytokine upregulation and effective antitumor activity in immune-competent mice-bearing tumors, and can enhance the curative effect of anti-PD1 monotherapy (26). In our study, patients with stage IB-IIIB NSCLC were treated with platinum-based NACT preoperatively, and we examined the expression of immune markers related to the cGAS-STING pathway and the density of tumor-infiltrating lymphocytes in tumor tissues before and after NACT, respectively. The results showed that the cytoplasmic levels of STING and IFN-β protein, PD-L1 on the surface of tumor cells increased after NACT, and the density of infiltrating CD3^+^ and CD8^+^ TILs in the tumor interstitium increased. Our findings suggest the effect of chemotherapy on some immune indicators in TME, thus adding to the rationale for neoadjuvant chemotherapy combined with immunotherapy.

## 5 Conclusions

NACT upregulated STING, PD-L1 and IFN-β protein expression in cGAS-STING pathway in NSCLC and increased CD3^+^ lymphocyte and CD8^+^ lymphocyte infiltration, suggesting the potential of NACT for sensitizing immunotherapy. In addition, CD3^+^ TILs infiltration changes after NACT, ypTNM stage and ypN stage were independent prognostic factors in DFS in NSCLC patients receiving NACT. The infiltration of CD3+ TILs changes and ypN stage were also independent prognostic factor for OS.

## Data availability statement

The original contributions presented in the study are included in the article/**Supplementary Material**. Further inquiries can be directed to the corresponding authors.

## Ethics statement

The studies involving humans were approved by the ethics committee of the First Affiliated Hospital of Xi’an Jiaotong University (No. XJTU1AF2021LSK-256). The studies were conducted in accordance with the local legislation and institutional requirements. The participants provided their written informed consent to participate in this study.

## Author contributions

HG: Data curation, Formal analysis, Investigation, Visualization, Writing – original draft, Writing – review & editing. XNZ: Data curation, Formal analysis, Visualization, Writing – original draft, Writing – review & editing. MR: Investigation, Software, Writing – review & editing. AJ: Software, Visualization, Writing – review & editing. NL: Software, Visualization, Writing – review & editing. JW: Writing – review & editing. XQZ: Writing – review & editing. XL: Writing – review & editing. ZR: Writing – review & editing. TT: Writing – review & editing. XF: Funding acquisition, Resources, Supervision, Writing – review & editing. YY: Conceptualization, Funding acquisition, Resources, Writing – review & editing.

## Supplementary material

Supplementary Figure 1 Kaplan-Meier curves of STING, IFN-β, PD-L1 IHC score and CD3^+^, CD8^+^ TILs density changes with disease free survival (DFS) and overall survival (OS) without significant statistical difference.

Supplementary Figure 2 Kaplan-Meier curves of disease free survival (DFS) based on pathological response **(A)** and pathological types **(B)**. Kaplan-Meier curves of overall survival (OS) based on pathological response.
