# Supplementary figures and images for: Platinum-based neoadjuvant chemotherapy upregulates STING/IFN pathway expression and promotes TILs infiltration in NSCLC
Source: Front Oncol. 2024 Feb 15;14:1346225. doi: 10.3389/fonc.2024.1346225 (PMC10902162; doi:10.3389/fonc.2024.1346225)

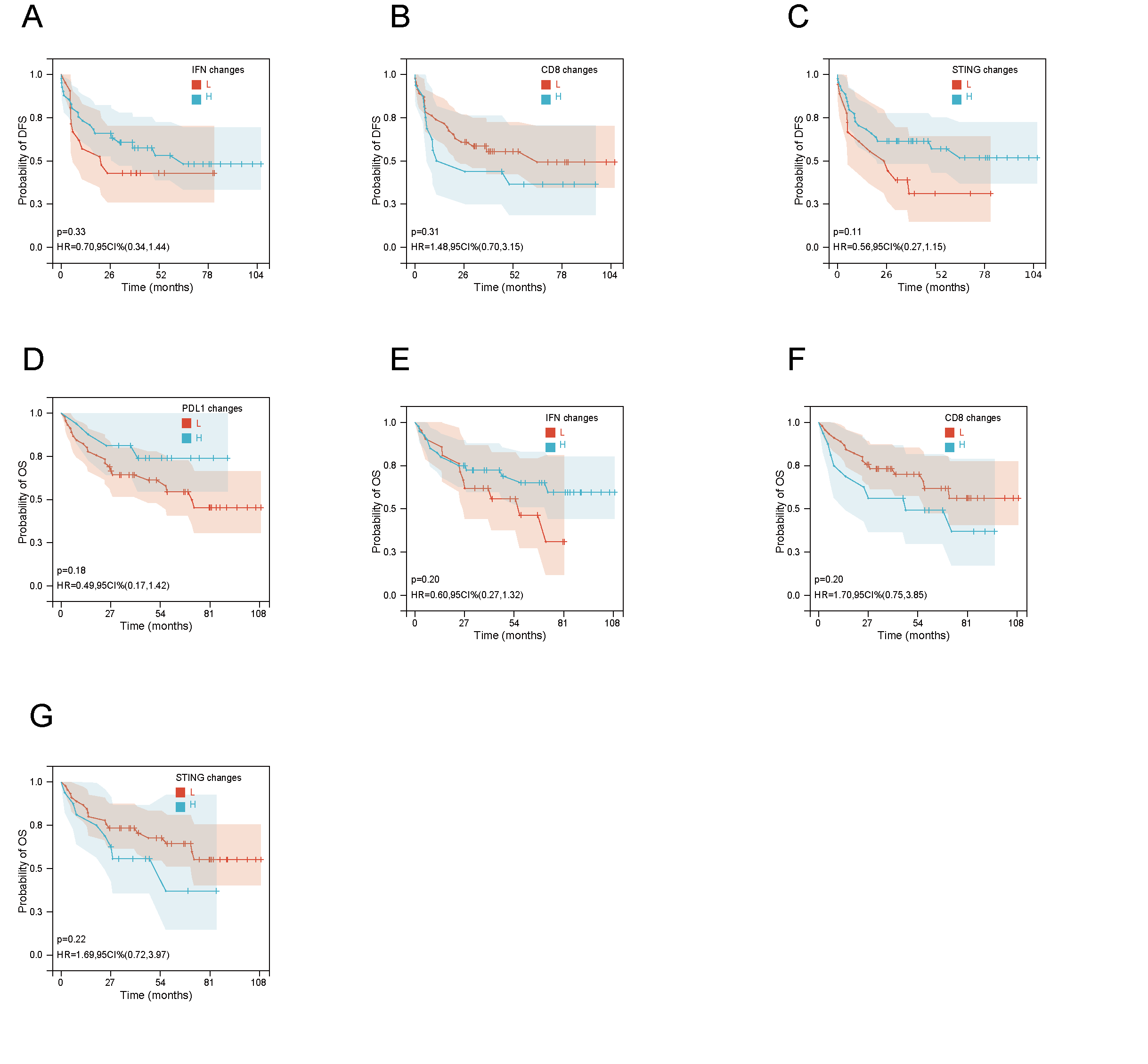

Supplement: Supplementary Figure 1 — Kaplan-Meier curves of STING, IFN-β, PD-L1 IHC score and CD3+, CD8+ TILs density changes with disease free survival (DFS) and overall survival (OS) without significant statistical difference. [file Image_1.tif]

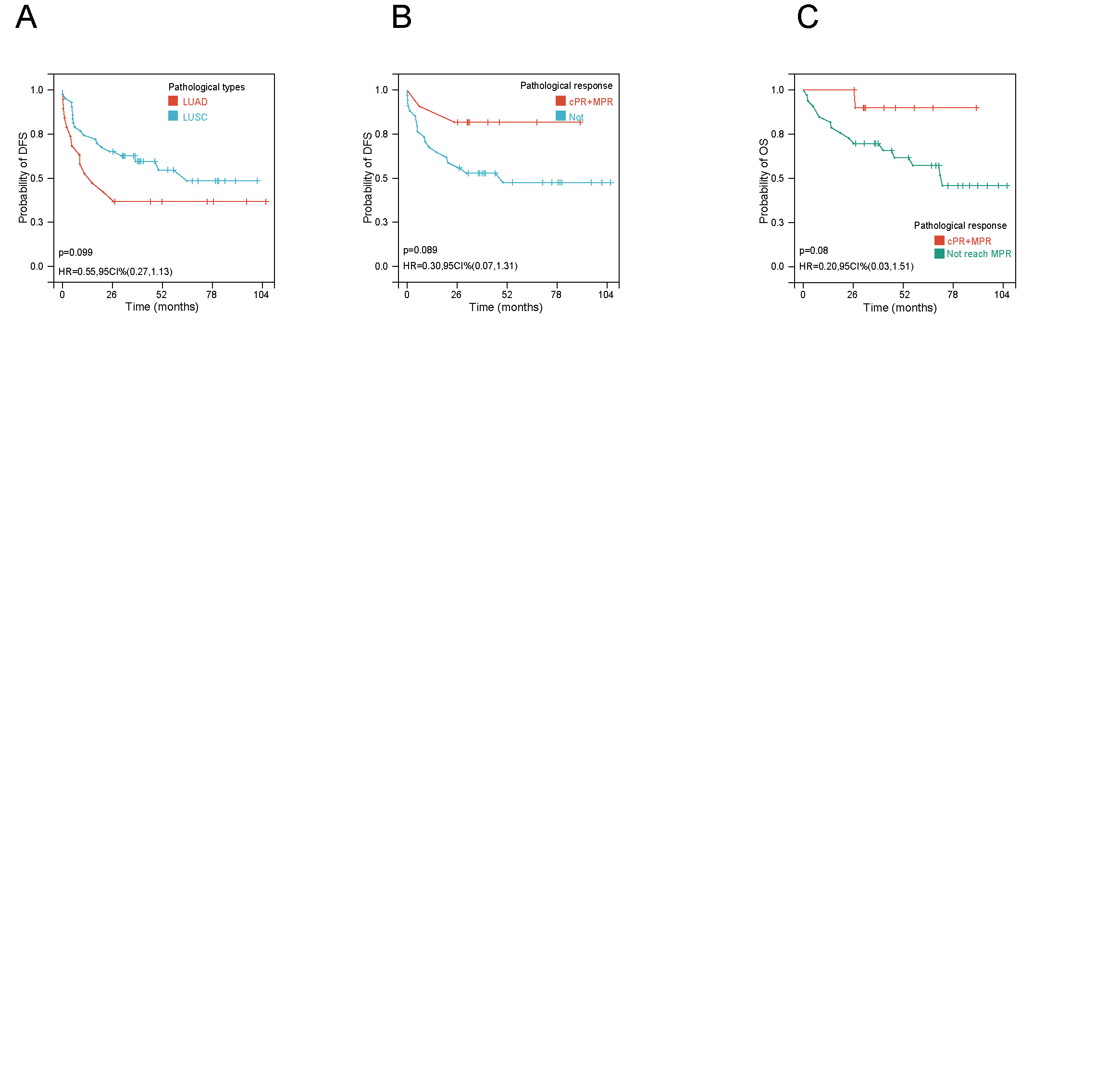

Supplement: Supplementary Figure 2 — Kaplan-Meier curves of disease free survival (DFS) based on pathological response (A) and pathological types (B). Kaplan-Meier curves of overall survival (OS) based on pathological response. [file Image_2.tif]
